# Diabetes risk loci-associated pathways are shared across metabolic tissues

**DOI:** 10.1186/s12864-022-08587-5

**Published:** 2022-05-14

**Authors:** Gerard A. Bouland, Joline W. J. Beulens, Joey Nap, Arno R. van der Slik, Arnaud Zaldumbide, Leen M. ’t Hart, Roderick C. Slieker

**Affiliations:** 1 Department of Cell and Chemical Biology, Leiden University Medical Center, Einthovenweg 20, 2333ZC Leiden, the Netherlands; 2 Department of Epidemiology and Data Science, Amsterdam UMC, Location VUMC, Amsterdam Public Health Institute, Amsterdam, the Netherlands; 3 Julius Center for Health Sciences and Primary Care, University Medical Center Utrecht, Utrecht, the Netherlands; 4 Department of Immunohematology and Blood Transfusion, Leiden University Medical Center, Leiden, The Netherlands; 5 Molecular Epidemiology Section, Department of Biomedical Data Sciences, Leiden University Medical Center, Leiden, the Netherlands

**Keywords:** Type 2 diabetes, SNPs, Pathways, Metabolism

## Abstract

**Aims/hypothesis:**

Numerous genome-wide association studies have been performed to understand the influence of genetic variation on type 2 diabetes etiology. Many identified risk variants are located in non-coding and intergenic regions, which complicates understanding of how genes and their downstream pathways are influenced. An integrative data approach will help to understand the mechanism and consequences of identified risk variants.

**Methods:**

In the current study we use our previously developed method CONQUER to overlap 403 type 2 diabetes risk variants with regulatory, expression and protein data to identify tissue-shared disease-relevant mechanisms.

**Results:**

One SNP rs474513 was found to be an expression-, protein- and metabolite QTL. Rs474513 influenced LPA mRNA and protein levels in the pancreas and plasma, respectively. On the pathway level, in investigated tissues most SNPs linked to metabolism. However, in eleven of the twelve tissues investigated nine SNPs were linked to differential expression of the ribosome pathway. Furthermore, seven SNPs were linked to altered expression of genes linked to the immune system. Among them, rs601945 was found to influence multiple *HLA* genes, including *HLA-DQA2*, in all twelve tissues investigated.

**Conclusion:**

Our results show that in addition to the classical metabolism pathways, other pathways may be important to type 2 diabetes that show a potential overlap with type 1 diabetes.

**Supplementary Information:**

The online version contains supplementary material available at 10.1186/s12864-022-08587-5.

## Introduction

Several large genome-wide association studies (GWASs) have been performed to understand the genetic drivers of type 2 diabetes (T2D). The most recent GWAS in almost one million people identified 403 variants [1–3]. While for some risk variants the underlying mechanisms are relatively well understood, for most the mechanisms are largely unclear. In an attempt to elucidate such mechanisms, previous studies have undertaken efforts to integrate data from public repositories or perform functional follow-up of loci [1, 4]. For example, a recent study used public data to assign scores to genes near 101 T2D risk variants to identify the causal genes [4]. Most studies have used the precalculated *cis* expression quantitative trait loci (eQTLs) from the GTEx project [4, 5]. GTEx provides eQTLs in a one Mb region around transcription start sites of genes, which could lead to missed eQTLs of variants in intergenic regions or with more distant genes. In addition, most studies, focused on a single tissue rather than considering all diabetes-relevant tissues. Finally, only few studies used other layers of information available in public data repositories such as DNA methylation-, miRNA -, protein-QTLs, chromatin interactions, chromatin state segmentations and transcription factor binding sites and expression data.

In the current study, we investigate 403 previously identified T2D-associated single nucleotide polymorphisms (SNPs) to gain insight into the pathways under influence of T2D risk variants. We overlap SNPs in multiple tissues and molecular levels, including chromatin state segmentations, multiple QTL modalities, including *cis* and *trans* eQTLs. Instead of focusing only on the eQTLs we investigate co-expression networks of eQTLs and look at the effects of eQTL shared by tissues. Our results confirm pathways known in T2D including metabolic pathways, but also identify potential other pathways including the ribosome and auto-immunity pathways.

## Results

**Fig. 1:**
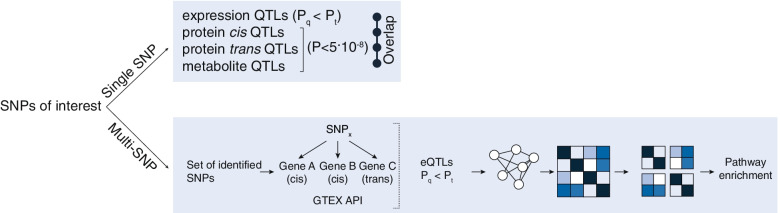
Setup of the current study

Four hundred three previously published T2D-associated SNPs [1] were investigated both individually and together (Fig. 1).

### The *SLC22A3* locus influences *SLC22A3* and *LPA* gene expression and plasma LPA protein levels

**Fig. 2:**
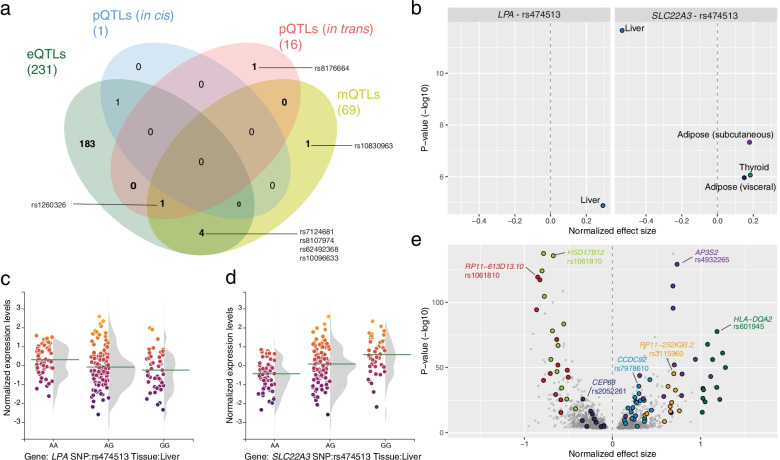
Tissue shared eQTLs for diabetes-associated SNPs.** a** Overlap between expression, protein (cis), protein (trans) and metabolite QTLs. **b** QTL effects of rs474513 on SLC22A3 and LPA** c** Effect of rs474513 on LPA mRNA expression in the liver. X-axis, genotype; y-axis, normalized expression levels. **d** Effect of rs474513 on SLC22A3 mRNA expression in the liver. X-axis, genotype; y-axis, normalized expression levels. **e** Volcano plot of all the 1619 calculated eQTL—eGene pairs, with the tissue-shared eQTL highlighted. X-axis, normalized effect size; y-axis, *P*-value. Dots with similar colors represent the same eQTLs in multiple tissues

SNPs were compared to expression QTLs (eQTLs), protein QTLs (*cis* and *trans* pQTLs) and metabolite QTLs (mQTLs). Among the 403 SNPs, there were 189 eQTLs across the 12 metabolic tissues investigated (Table S2), 1 *cis*-pQTL, 2 *trans*-pQTLs and 6 mQTLs. One SNP rs1260326 was an eQTL, pQTL and mQTL (Fig. 2a, Table S3). There were multiple eQTLs in multiple tissues. Rs1260326-C was associated with lower *SNX17* mRNA levels in the muscle (β = -0.32, *P* = 1.29·10^–32^, Table S3) and lower mRNA levels of *NRBP1* in subcutaneous and visceral fat, whole blood, intestine, transverse colon. Of note, based on the H4 posterior probability (H4 PP), type 2 diabetes and *NRBP1* (H4 PP ≥ 0.9) shared causal variants in five tissues, while the H4 PP for *SNX17* was much lower (0.483). Rs1260326 has been associated with Glucokinase Regulatory Protein, but only in thyroid, this SNP was an eQTL with GCKR (β = 0.22, *P* = 1.84·10^–8^). In plasma, rs1260326-T was associated with upregulated alanine and multiple lipid levels, including very-low-density lipoproteins (VLDL) and triacylglycerols levels. Finally, *in trans* rs1260326-T was associated with higher levels of plasma Insulin-like growth factor-binding protein 1 (IGFBP1, *P* = 2.42·10^–13^, H4 PP = 0.873), Kallikrein B1 (KLKB1, P = 2.13·10^–10^), although the latter showed a very low H4 PP (4.23·10^–46^). KLKB1 is involved in blood coagulation and IGFBP1 is involved in metabolism.

One SNP rs474513 was an eQTL and a *cis* pQTL (Fig. 2a, Table S4). In the liver, rs474513-A was an eQTL with apolipoprotein(a) (*LPA*, β = 0.29, *P* = 1.29·10^–5^, Figs. 2a and c). The A-allele of rs474513 gives an increased type 2 diabetes risk [1]. In plasma, rs474513-A influenced LPA protein levels (β = 0.23, *P* = 8.27·10^–37^). In multiple tissues, rs474513-A was an eQTL for *SLC22A3* with an increase in expression, interestingly the liver was the sole tissue with decreased expression (β = -0.53, *P* = 2.23·10^–12^, Fig. 2d). Interestingly *SLC22A3* encodes the organic cation transporter 3 (OCT3) which is involved in metformin transport [6]. Of note, the H4 PP do however suggest that these QTLs do not share causal variants with type 2 diabetes, limiting the implications and interpretation of these results.

The 189 SNPs that were identified as eQTL influenced the expression of 556 genes with in total 1619 eQTL-eGene pairs across investigated tissues (Fig. 2e, Table S2). Several diabetes risk variants increased the expression of genes in multiple tissues, including *AP3S2* (rs4932265-T), *CCDC92* (rs7978610-G), *HLA-DQA2* (rs601945-G) and a lncRNA *RP11-252K23.2* (Table S2, Fig. S1, rs3115960-G). Genes that showed decreased expression with diabetes-risk alleles included *CEP68* (rs2052261-A, rs2249105-A), *HSD17B12* (rs1061810-A) and the long non-coding RNA (lncRNA) *RP11-613D13.10* (rs1061810-A). *HLA-DQA2* is involved in multiple disease- and immune response-related pathways [7]. The two SNPs that influenced the expression of Centrosomal Protein 68 (*CEP68*) were in LD (*r*^2^ = 0.798). *CEP68* is involved in centrosome cohesion [8]. *AP3S2* is part of the AP-3 complex which is associated with the Golgi region and involved in vesicle transport [9]. *HSD17B12* encodes Hydroxysteroid 17-Beta Dehydrogenase 12, which is involved in synthesis of fatty acids [7]. Rs1061810 influenced both the expression of *HSD17B12* and *RP11-613D13.10*, where the latter is likely the *HSD17B12* antisense, given that the expression of both transcripts was strongly correlated (Spearman’s ρ = 0.65–0.90, Fig. S2). *CCDC92* has been associated with impaired adipogenesis [10]. Of note, based on the posterior probability, only *HSD17B12*, *R11-613D13.10* shared causal variants with type 2 diabetes (Table S2).

### Type 2 diabetes-associated eQTLs link to ribosome and autoimmunity pathways

**Fig. 3:**
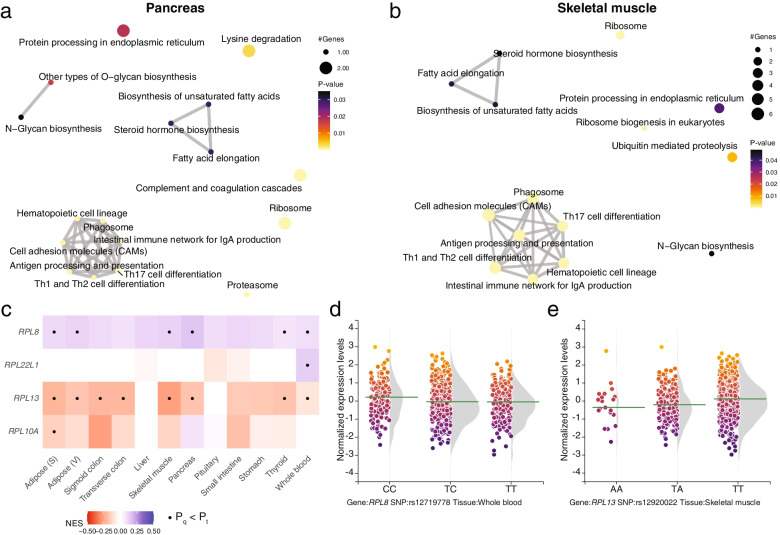
Diabetes eQTLs are linked to metabolic pathways, including the ribosome pathway **a**-**b**. Enriched pathways in the pancreas (**a**) and the muscle (**b**). Grey lines between nodes indicate overlap between eGenes. **c** eQTLs associated with the Ribosome pathway. X-axis tissues investigated, y-axis eGenes and eQTLs. Circles indicate significant *P*-values. **d** Effect of rs12719778 on RPL8 mRNA expression in whole blood. X-axis, genotype; y-axis, normalized expression levels. **e** Effect of rs12920022 on RPL13 mRNA expression in skeletal muscle. X-axis, genotype; y-axis, normalized expression levels

Next, eQTLs were investigated in more detail to find common and tissue-specific pathways across tissues. The strongest enriched Kyoto Encyclopedia of Genes and Genomes (KEGG) pathways were not those linked to metabolism (Table S5). Instead, the strongest enriched pathway was the *Ribosome*, which was enriched in six of the twelve tissues (Table S5, Fig. S3). Also when the enrichment was based on REACTOME instead of KEGG, the top enriched pathway was *Eukaryotic Translation Elongation*, in which the ribosomes play a key role (Table S6). The ribosome pathway was followed by pathways that are related to immunity and all these pathways were driven by the same set of genes as indicated by the lines connecting pathways (Fig. 3a-b, Fig. S4). Immune-related pathways were identified in 10 of the 12 tissues. For the ribosome pathway the number of eQTLs varied across tissues from a single in pituitary up to nine SNPs in subcutaneous fat. Across the twelve tissues investigated, two eQTL-eGene pairs were most consistently observed, that is rs12719778-T/*RPL8* and rs12920022-A/*RPL13* (Fig. 3c). The biggest effect size of rs12719778-T/*RPL8* was observed in whole blood (NES = -0.10, *P* = 8.46·10^–13^, Fig. 3d), while for rs12920022-A/*RPL13* the largest normalized effect size was observed in skeletal muscle (NES = -0.26, *P* = 3.36·10^–25^, Fig. 3e). The pathways that were identified in less tissues were those related to metabolism, for example in seven tissues *Biosynthesis of unsaturated fatty acids* which was driven by the effect of rs1061810 on *HSD17B12* (Table S5).

### Multiple diabetes-associated SNPs link to genes linked to autoimmunity

**Fig. 4:**
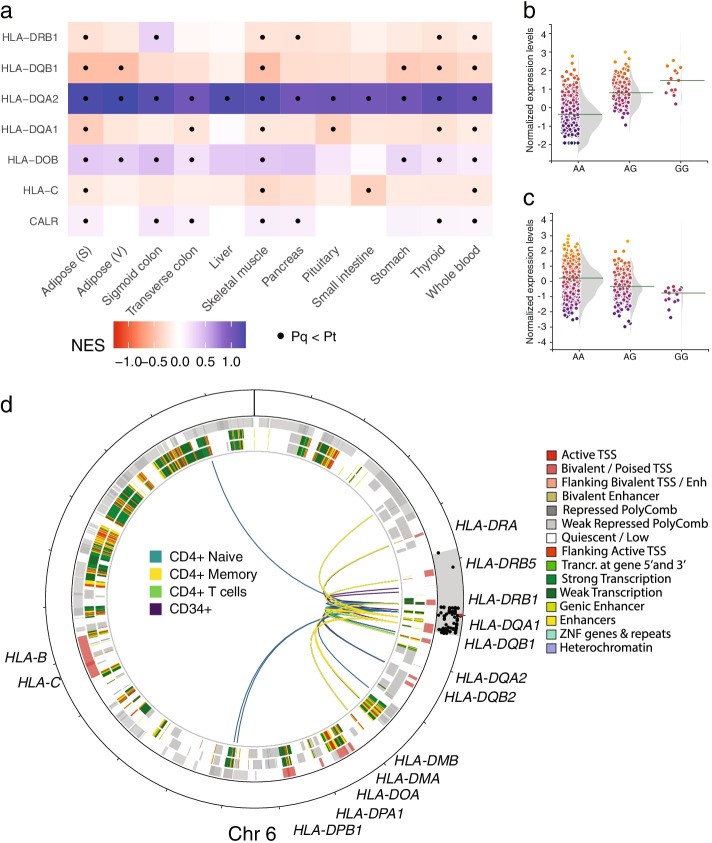
Diabetes eQTLs linked to immune system-related pathways. **a **eQTLs associated with the Antigen processing and presentation pathway driven by rs601945. X-axis tissues investigated, y-axis eGenes and eQTLs. **b **Effect of rs601945 on HLA-DQA2 mRNA expression in the skeletal muscle. X-axis, genotype; y-axis, normalized expression levels. **c** Effect of rs12719778 on HLA-DQB1 mRNA expression in the skeletal muscle. X-axis, genotype; y-axis, normalized expression levels. **d **Circos plot of the rs601945 locus. Chromatin interactions in blood cells (CD34^+^, CD4^+^ memory, CD4^+^ naïve and CD4^+^ T) of the region that is in LD (r^2^ ≥ 0.8, lead SNP is shown in red) with rs601945 are shown as colored arcs. Chromatin states are shown for CD4^+^ memory (inner-ring) and CD4^+^ naïve cells respectively (middle-ring). Genes are shown in the outer ring in gray and red

The immune-related pathways were generally enriched based on the same gene set in all tissues investigated (Fig. 3a-b, Fig. S4**)**. Across tissues, the *Antigen processing and presentation* pathway was the strongest enriched pathway of the immune-related pathways (Table S5) which involved in total seven eQTLs and 18 genes. Comparison of the KEGG enrichment with that based on REACTOME resulted also in a strong enrichment for antigen presentation pathways, including *PD-1 signaling* and *MHC class II antigen presentation* (Table S6)*.* Rs601945 was the eQTL that influenced the most genes involved (Fig. 4a). Rs601945 is a QTL in the HLA region, with the strongest positive effect on *HLA-DQA2* in the skeletal muscle (NES = 1.19, *P* = 2.50·10^–78^, Fig. 4b)*,* while rs601945 had a negative effect on *HLA-DQB1* with the strongest effect, again, in skeletal muscle (NES = -0.50, *P* = 1.18·10^–16^*,* Fig. 4c). Based on histone modifications across tissues, the HLA locus was generally quiescent, except for blood cells where in multiple blood cell types enhancers were found (Fig. S5). In line with this, multiple chromatin interactions were observed in blood cells (CD34^+^, CD4^+^ memory, CD4^+^ naïve and CD4^+^ T-cells, Fig. 4d), 40 of which are interactions with loci located in HLA genes, with the top genes *HLA-DQA1* (19 interactions) and *HLA-DQB1* (13 interactions, Fig. 4d). Rs601945 has previously also been identified as a risk factor for autoimmunity diseases, including type 1 diabetes (*P* = 5.72·10^–80^), ulcerative colitis (*P* = 1.05·10^–28^) and inflammatory bowel disease (*P* = 2.34·10^–24^) based on data from the T1D portal (https://t1d.hugeamp.org).

Moreover, rs601945 was associated with blood cell counts of neutrophils (*P* = 2.53·10^–112^), eosinophils (*P* = 1.36·10^–37^) and monocytes (*P* = 1.70·10^–15^).

## Discussion

In this study, we investigated 403 T2D-associated SNPs in more detail. We show that variants can have effects across multiple molecular layers, including gene, protein and metabolite levels. Moreover, risk variants are associated with altered expression of the same genes in multiple tissues, including *HSD17B12*, *AP3S2*, *HLA-DQA2*. This was also reflected in observed enriched pathways, where the same pathways were influenced by genetic risk for diabetes across tissues, including antigen processing, ribosome, proteasome and protein processing in endoplasmic reticulum.

One T2D-associated variant, intronic of *SLC22A3* was both an expression- and protein-QTL. The T2D risk allele A of rs474513 was associated with higher expression of *LPA* expression in the liver and higher protein levels in plasma. High LPA levels have been associated with higher risk on cardiovascular disease events in people with diabetes [11]. In addition to LPA, rs474513-A was associated with higher expression of *SLC22A3* (OCT3).

Several T2D-associated risk-alleles showed tissue-shared eQTL effects, including *AP3S2*, *CCDC92*, *HLA-DQA2, CEP68*, *HSD17B12* and two lncRNAs *RP11-613D13.10* and *RP11-252K23.2*. Both *AP3S2* and *HSD17B12* have previously been found in relation to T2D, but in limited number of tissues. *AP3S2* in human pancreatic islets [12] and *HSD17B12* in adipose, liver, muscle tissue and whole blood [3], which are relevant for T2D [13]. One of the strongest observed effects was between rs4932265 and *AP3S2*. *AP3S2* is a subunit of the AP-3 complex which is involved in budding of vesicles from the Golgi membrane [9]. *AP3S2* has been linked to T2D in six different GWASs [14–19] investigating various populations (South Asian, Japanese and European ancestry) and with four different SNPs, three of which (rs12912009, rs2028299, rs8031576) are in LD (*r*^*2*^ ≥ 0.80) with rs4932265. In the current study we observed that *AP3S2* has a higher expression in the twelve tissues in individuals carrying the risk allele of rs4932265. Despite increasing evidence for the role *AP3S2* in T2D susceptibility it remains unclear how *AP3S2* is involved, although there is some evidence pointing at a beta-cell defect [20]. Nonetheless, our data suggest that the effect may be more tissue-shared than limited to one cell type.

For *HSD17B12*, we show that the diabetes-risk risk allele of rs1061810 was associated with a lower expression in all twelve tissues investigated. The *HSD17B12* gene encodes a bifunctional enzyme involved in the biosynthesis of estradiol and the elongation of very long chain fatty acids [21]. This result corroborates the finding that *HSD17B12* expression is downregulated in the adipose tissue of insulin-resistant subjects [22] and that *HSD17B12* plays a role in adipogenesis [23]. Rs1061810 was also associated with altered expression of *RP11-613D13.10* and which is the antisense of *HSD17B12*. The role of the lncRNAs *RP11-613D13.10* and *RP11-252K23.2* is not clear. Furthermore, we observe in the current study that based on the H4 PP causal variants were shared between type 2 diabetes and *HSD17B12* / *R11-613D13.10*.

On the pathway level as expected most SNPs linked to metabolic pathways. The metabolic pathways as curated by KEGG [7] consists of 1489 genes and is an encompassing term for all pathways that are involved in metabolism. Our results show that SNPs that are directly linked to metabolism do not influence a single metabolic process but are scattered among various metabolic pathways. Due to this dispersion of SNPs between numerous pathways it remains difficult to assign groups of SNPs to specific processes in specific tissues. This together with the variety of pathways to which SNPs are mapped shows that T2D has a lot of different points of engagement through which it can originate and progress, which is accordance with the heterogeneous nature of T2D [24].

In eleven of the twelve tissues, eQTLs were enriched for the ribosome pathway. Genetic susceptibility to T2D has previously not been linked to a decreased expression of ribosomal genes, although the association between ribosomal content and T2D has extensively been studied [25–27]. Insulin and ribosomal content are tightly connected, where insulin stimulates the synthesis of ribosomal proteins in various tissues [28, 29] and a loss of ribosomal proteins is associated with an inhibition of AKT phosphorylation activity/insulin pathway [30].

Multiple enriched pathways linked to immunity and the eGenes in these pathways were mainly from the HLA class. Rs601945 was the key SNP in these pathways as it influences the expression of multiple HLA genes across tissues. HLA genes have previously been associated with T2D [3, 17], however, our results reveal that the effects are widespread as its association with altered expression of various HLA genes was observed in all investigated tissues. Interestingly, while the HLA region represents the highest risk for T1D [31], our results are pointing to a connection between *HLA-DQA2* and T2D. Rs601945 has also been associated with other diseases where autoimmunity plays a key role, including type 1 diabetes, ulcerative colitis, inflammatory bowel disease. Moreover, rs601945 is strongly associated with blood cell counts of neutrophils, eosinophils and monocytes. These results suggest that the r601945 is associated with autoimmunity. These results also support previous results that diabetes could also be considered as a continuum rather than two separate diseases with overlap [32, 33]. Our data support that T2D has an immuno-metabolic component involving, like T1D, members of both innate and adaptive immune response. In addition, our pQTL analyses also highlighted immune response pathways. Also, in a previous study in blood of persons with T2D we found that HbA1c is associated with altered expression of immune response-related genes [34].

Strength of our study is the hypothesis-free approach in multiple tissues, which allowed us to investigate tissue-shared effects of T2D-associated SNPs. A limitation of our study is that we mostly rely on eQTLs where we do not know whether the observed changes in expression also translate to changes in protein levels. A second limitation is that we use QTL data from different sets of individuals, while ideally one would use regulatory, expression and protein data from the same set of individuals. Third, we rely on data from European descent, which limits the generalizability to other ethnicities. Fourth, a limitation is that in the current study we do not validate our findings in vitro, which is required to fully understand the observed results. Finally, the pQTLs included in the current study were only measured in plasma and are therefore not necessarily representative of the pQTLs within tissues.

## Conclusion

Altogether, our data show biological processes that are subject to genetic influences. We show that they are not necessarily limited to single tissues but are shared across diabetes-relevant tissues. Our findings highlight the importance of an integrative tissue-wide approach where risk loci for T2D are not only seen as individual risk factors but also as a network of risk factors that may play a role across tissues.

## Methods

Four hundred three SNPs from the GWAS summary statistics reported by Mahajan et al. [1] were extracted. SNPs plus those in LD were used in subsequent analyses, where LD was defined as *r*^2^ ≥ 0.80 around the respective variants. The SNPs were analyzed using our previously published package CONQUER [35]. Both the single SNP mode and multi-SNP mode were used to analyze the SNPs of interest. For the single SNP analysis we extracted data from relevant databases using our R-package CONQUER, including expression quantitative trait loci (eQTLs) *in cis* and *trans*, DNA methylation-, protein-QTLs, chromatin interactions, chromatin state segmentations, transcription factor binding sites and expression data [35].

### QTL data

eQTLs were obtained from GTEx based on data from mainly European descent. All lead SNPs were tested against all genes (eGenes) *in cis* and *trans* on the GTEx API. eGenes are genes under influence of an eQTL. Trans-eQTLs were those more distant from the SNP but on the same chromosome and the tested region was defined by the range of the predicted chromosomal interactions. SNPs were considered significant if the P-value was below the GTEx threshold. Effect sizes in figures and tables are those for the effect allele of T2D relative to the alternative allele based on an additive model. The latter threshold is defined as the empirical P-value of the gene closest to the 0.05 FDR threshold. Significant protein-QTLs (pQTLs, *P* < 5·10^–8^) obtained from Yao et al. [36] and filtered for the 403 lead SNPs plus SNPs in LD with those SNPs. QTLs in Yao et al. [36] have been identified in people from European descent. Significant metabolite QTLs (mQTLs, *P* < 5·10^–8^) were obtained Gallois et al. [37] based on a Finnish population.

### GTEx gene expression data

Expression data was obtained from GTEx V8 for tissues relevant for the etiology of T2D, including subcutaneous and visceral fat, sigmoid- and transverse colon, liver, skeletal muscle, pancreas, pituitary, terminal ileum of the small intestine, stomach, thyroid and whole blood. Of the included tissues, sample sizes range from *N* = 187 (terminal ileum) to *N* = 803 (skeletal muscle). The percentage males was relatively higher (63.1%—72.1%, Table S1) with the majority middle-aged (50–69 years, Table S1).

### Colocalization analysis

To compare whether two traits shared a common causal variant, a colocalization analysis was performed using the R-package *coloc*. The measure for a single shared causal variant is the H4 posterior probability, where a H4 PP larger than 0.9 indicates a single causal variant shared by two traits. In the current study, we use the colocalization to compare the type 2 diabetes SNPs to the protein and expression QTLs.

### Multi-SNP analysis

Modularization and pathway enrichment for included tissues was performed on all significant eQTLs (see above). Genes co-expressed with eGenes were identified (*ρ* ≥|0.90|*,* Spearman's rank correlation coefficient). The latter was done to identify entire pathways that are under influence of one or more SNPs. The eGenes and co-expressed genes were hierarchical clustered [38, 39] based on pairwise distance between genes (*1 –* ρ). The number of modules within the clustered data was optimized by maximizing the globalSEmax of the gap statistic [40] using the *cluster* R package [41]. Modules of co-expressed genes and eGenes were tested for pathway enrichment based on KEGG [7] pathways. For each module, association with pathways was determined with Fisher’s exact test [42], which resulted in odds ratios and accompanying P-values on the association. If a module did not contain an eQTL or was not enriched for a pathway, it was omitted from the results. Figures were produced in and obtained from CONQUER or additionally made using the R-package *ggplot2* version 3.2.1. [43].

## Supplementary Information

**Additional file 1: Figure S1. a **Frequency of tissues in which a certain eGene was identified. X-axis, genes under influence of a diabetes SNP, y-axis, frequency of tissues in which the QTL was identified. **Figure S2.** Scatterplot of expression of RP11-613D13.10 versus the expression of HSD17B12. X-axis, expression of RP11-613D13.10; y-axis expression of HSD17B12.** Figure S3.** **a** Frequency of the number of times an enriched pathway was found across the twelve tested tissues. X-axis, pathway investigated; y-axis, frequency of tissues. Blue bars indicate pathways associated with HLA-genes. **Figure S4.** Relation between KEGG pathways identified in each of the tissues. Tissues include subcutaneous fat (a), visceral fat (b), sigmoid colon (c), transverse colon (d), pituitary (e), small intestine (f), stomach (g), thyroid (h), whole blood (i). **Figure S5.** Chromatin state segmentations for rs601945 in various tissues and cell lines (data from Epigenomics Roadmap). Plus-symbol indicates a SNP in LD and blood cell types are colored in blue. X-axis, location on the genome; y-axis cell type.

**Additional file 2: Table S1. **Characteristics of the individuals in the GTEX data.

**Additional file 3: Table S2. **eQTLs associated with T2D SNPs.

**Additional file 4: Table S3. **Protein, metabolite and expression QTLs associated with rs1260326.

**Additional file 5: Table S4. **Protein and expression QTLs associated with rs474513.

**Additional file 6: Table S5. **Enriched KEGG pathways based on T2D SNPs.

**Additional file 7: Table S6. **Enriched REACTOME pathways based on T2D SNPs.

## Data Availability

All data presented in the current manuscript are publicly available and implemented in the R-package CONQUER.db (https://github.com/roderickslieker/CONQUER.db). eQTLs and expression data were obtained from GTEx. Pathways were obtained from KEGG (https://www.genome.jp/kegg/).
