# Supplementary material for: Diabetes risk loci-associated pathways are shared across metabolic tissues
Source: BMC Genomics. 2022 May 14;23:368. doi: 10.1186/s12864-022-08587-5 (PMC9107144; doi:10.1186/s12864-022-08587-5)
Supplement: Supplementary file 1 — Additional file 1: Figure S1. a Frequency of tissues in which a certain eGene was identified. X-axis, genes under influence of a diabetes SNP, y-axis, frequency of tissues in which the QTL was identified. Figure S2. Scatterplot of expression of RP11-613D13.10 versus the expression of HSD17B12. X-axis, expression of RP11-613D13.10; y-axis expression of HSD17B12. Figure S3. a Frequency of the number of times an enriched pathway was found across the twelve tested tissues. X-axis, pathway investigated; y-axis, frequency of tissues. Blue bars indicate pathways associated with HLA-genes. Figure S4. Relation between KEGG pathways identified in each of the tissues. Tissues include subcutaneous fat (a), visceral fat (b), sigmoid colon (c), transverse colon (d), pituitary (e), small intestine (f), stomach (g), thyroid (h), whole blood (i). Figure S5. Chromatin state segmentations for rs601945 in various tissues and cell lines (data from Epigenomics Roadmap). Plus-symbol indicates a SNP in LD and blood cell types are colored in blue. X-axis, location on the genome; y-axis cell type. [file 12864_2022_8587_MOESM1_ESM.pdf]

Supplementary figures

a

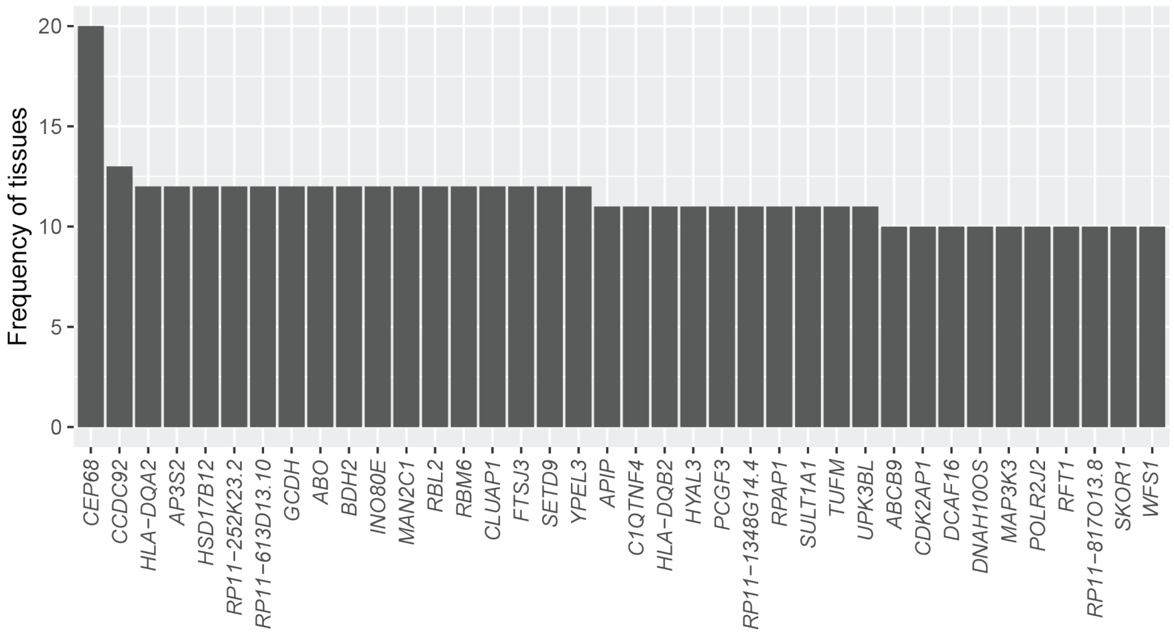

Figure S1. a Frequency of tissues in which a certain eGene was identified. X-axis, genes under influence of a diabetes SNP, y-axis, frequency of tissues in which the QTL was identified.

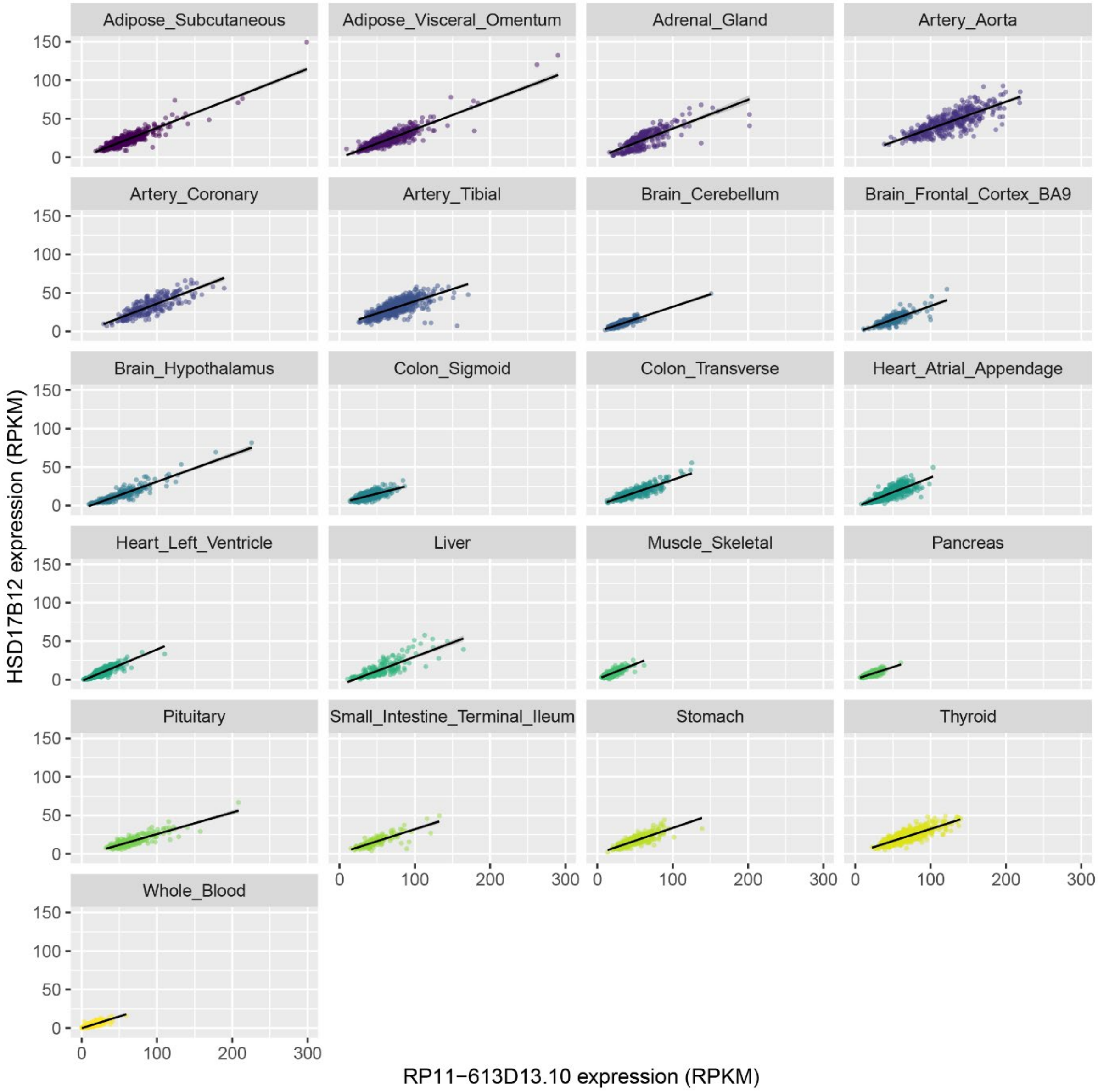

Figure S2. Scatterplot of expression of *RP11-613D13.10* versus the expression of *HSD17B12*. X-axis, expression of RP11-613D13.10; y-axis expression of *HSD17B12*.

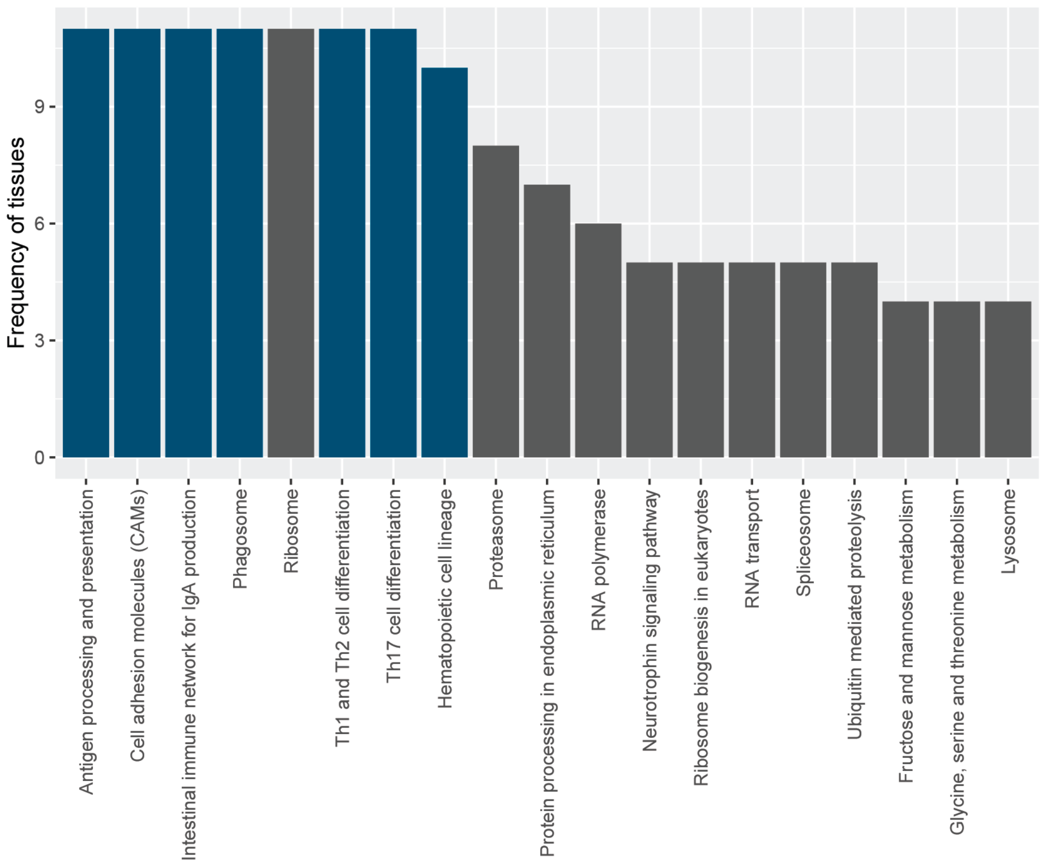

Figure S3. **a** Frequency of the number of times an enriched pathway was found across the twelve tested tissues. X-axis, pathway investigated; y-axis, frequency of tissues. Blue bars indicate pathways associated with HLA-genes.

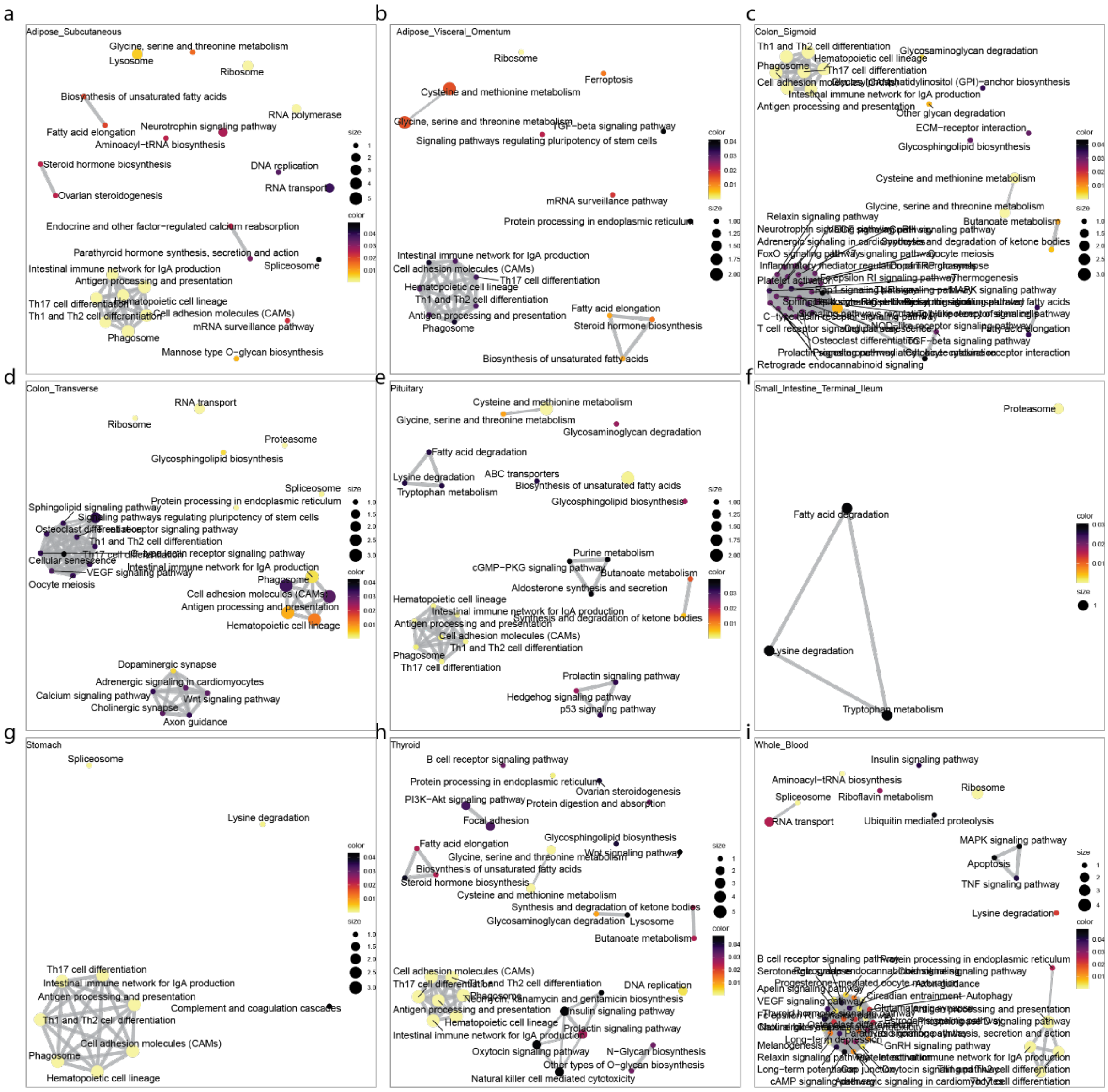

Figure S4 Relation between KEGG pathways identified in each of the tissues. Tissues include subcutaneous fat (a), visceral fat (b), sigmoid colon (c), transverse colon (d), pituitary (e), small intestine (f), stomach (g), thyroid (h), whole blood (i).

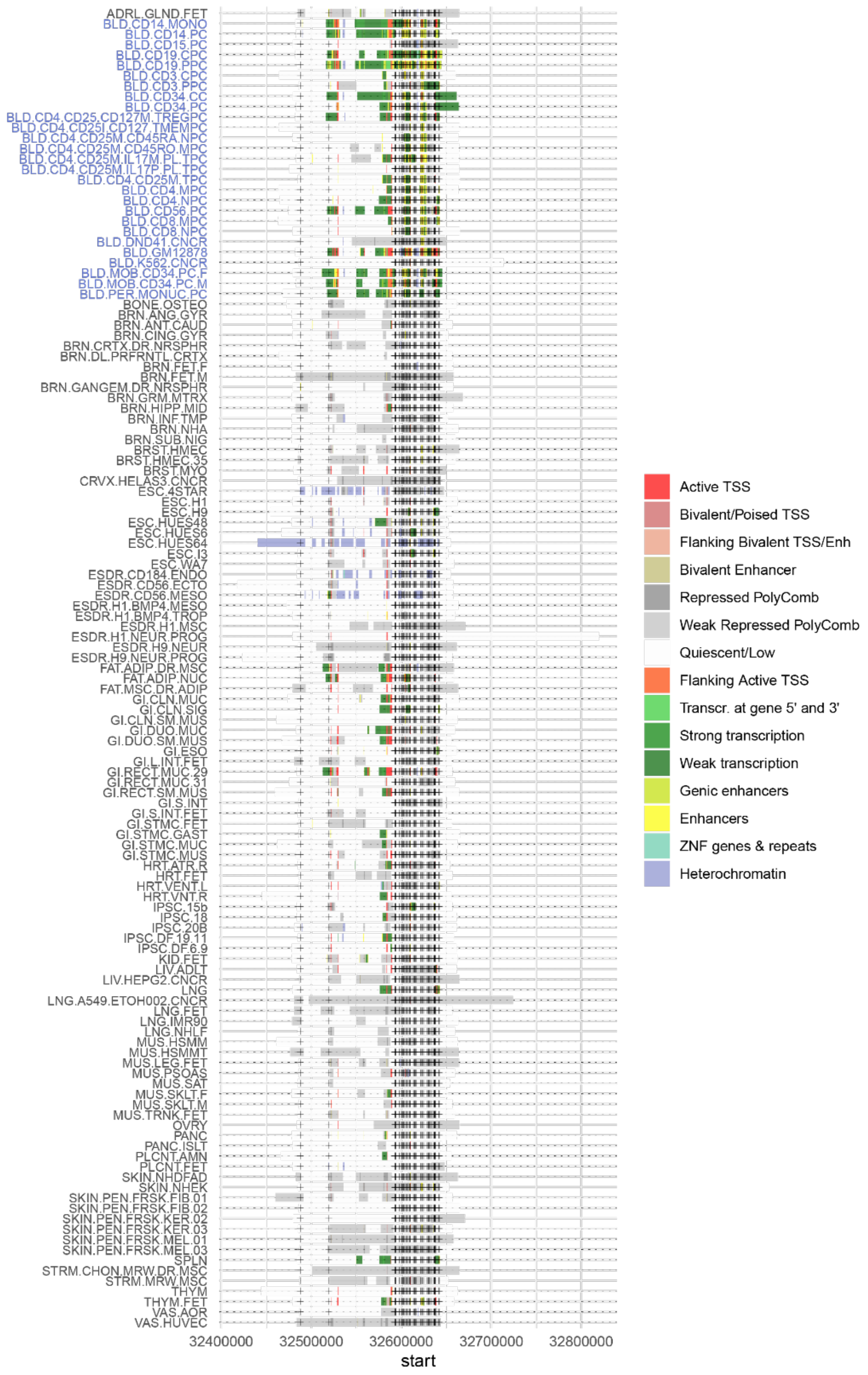

Figure S5. Chromatin state segmentations for rs601945 in various tissues and cell lines (data from Epigenomics Roadmap). Plus-symbol indicates a SNP in LD and blood cell types are colored in blue. X-axis, location on the genome; y-axis cell type.
