# Supplementary material for: Diabetes risk loci-associated pathways are shared across metabolic tissues
Source: BMC Genomics. 2022 May 14;23:368. doi: 10.1186/s12864-022-08587-5 (PMC9107144; doi:10.1186/s12864-022-08587-5)
Supplement: Supplementary file 2 — Additional file 2: Table S1. Characteristics of the individuals in the GTEX data. [file 12864_2022_8587_MOESM2_ESM.docx]

***Table S1. Characteristics of the individuals in the GTEX data***

|  |  |  | **Age** | | | | | | **Cause of Death** | | | | | |
| --- | --- | --- | --- | --- | --- | --- | --- | --- | --- | --- | --- | --- | --- | --- |
| **Tissue** | **N** | **Sex (male)** | **20-29** | **30-39** | **40-49** | **50-59** | **60-69** | **70-79** | **0** | **1** | **2** | **3** | **4** | **missing** |
| Adipose Subcutaneous | 663 | 445 (67,1 %) | 52 (7,8 %) | 58 (8,7 %) | 103 (15,5 %) | 213 (32,1 %) | 216 (32,6 %) | 21 (3,2 %) | 352 (53,1 %) | 21 (3,2 %) | 176 (26,5 %) | 32 (4,8 %) | 70 (10,6 %) | 12 (1,8 %) |
| Adipose Visceral Omentum | 541 | 371 (68,6 %) | 45 (8,3 %) | 44 (8,1 %) | 86 (15,9 %) | 184 (34 %) | 163 (30,1 %) | 19 (3,5 %) | 314 (58 %) | 16 (3 %) | 136 (25,1 %) | 25 (4,6 %) | 50 (9,2 %) | 0 (0 %) |
| Colon Sigmoid | 373 | 240 (64,3 %) | 36 (9,7 %) | 39 (10,5 %) | 54 (14,5 %) | 109 (29,2 %) | 123 (33 %) | 12 (3,2 %) | 236 (63,3 %) | 9 (2,4 %) | 73 (19,6 %) | 16 (4,3 %) | 39 (10,5 %) | 0 (0 %) |
| Colon Transverse | 406 | 259 (63,8 %) | 43 (10,6 %) | 47 (11,6 %) | 75 (18,5 %) | 136 (33,5 %) | 94 (23,2 %) | 11 (2,7 %) | 316 (77,8 %) | 4 (1 %) | 47 (11,6 %) | 14 (3,4 %) | 24 (5,9 %) | 1 (0,2 %) |
| Liver | 226 | 161 (71,2 %) | 7 (3,1 %) | 17 (7,5 %) | 35 (15,5 %) | 83 (36,7 %) | 79 (35 %) | 5 (2,2 %) | 86 (38,1 %) | 13 (5,8 %) | 93 (41,2 %) | 15 (6,6 %) | 18 (8 %) | 1 (0,4 %) |
| Muscle Skeletal | 803 | 543 (67,6 %) | 67 (8,3 %) | 65 (8,1 %) | 124 (15,4 %) | 255 (31,8 %) | 264 (32,9 %) | 28 (3,5 %) | 424 (52,8 %) | 31 (3,9 %) | 203 (25,3 %) | 46 (5,7 %) | 87 (10,8 %) | 12 (1,5 %) |
| Pancreas | 328 | 207 (63,1 %) | 29 (8,8 %) | 31 (9,5 %) | 66 (20,1 %) | 118 (36 %) | 79 (24,1 %) | 5 (1,5 %) | 275 (83,8 %) | 3 (0,9 %) | 28 (8,5 %) | 7 (2,1 %) | 15 (4,6 %) | 0 (0 %) |
| Pituitary | 283 | 204 (72,1 %) | 9 (3,2 %) | 8 (2,8 %) | 24 (8,5 %) | 87 (30,7 %) | 138 (48,8 %) | 17 (6 %) | 13 (4,6 %) | 21 (7,4 %) | 159 (56,2 %) | 27 (9,5 %) | 63 (22,3 %) | 0 (0 %) |
| Small Intestine Terminal Ileum | 187 | 120 (64,2 %) | 28 (15 %) | 22 (11,8 %) | 35 (18,7 %) | 56 (29,9 %) | 42 (22,5 %) | 4 (2,1 %) | 174 (93 %) | 2 (1,1 %) | 7 (3,7 %) | 1 (0,5 %) | 3 (1,6 %) | 0 (0 %) |
| Stomach | 359 | 227 (63,2 %) | 44 (12,3 %) | 39 (10,9 %) | 64 (17,8 %) | 128 (35,7 %) | 79 (22 %) | 5 (1,4 %) | 301 (83,8 %) | 3 (0,8 %) | 35 (9,7 %) | 5 (1,4 %) | 14 (3,9 %) | 1 (0,3 %) |
| Thyroid | 653 | 434 (66,5 %) | 47 (7,2 %) | 51 (7,8 %) | 110 (16,8 %) | 211 (32,3 %) | 212 (32,5 %) | 22 (3,4 %) | 358 (54,8 %) | 24 (3,7 %) | 172 (26,3 %) | 32 (4,9 %) | 65 (10 %) | 2 (0,3 %) |
| Whole Blood | 755 | 501 (66,4 %) | 68 (9 %) | 68 (9 %) | 113 (15 %) | 234 (31 %) | 249 (33 %) | 23 (3 %) | 412 (54,6 %) | 29 (3,8 %) | 188 (24,9 %) | 36 (4,8 %) | 77 (10,2 %) | 13 (1,7 %) |

*Data characteristics of the investigated tissues classified by age and separately by cause of death. Cause of death based on 4-point Hardy-Scale: 0) Cases on ventilator before death 1) violent and fast death, i.e. accident 2) fast death of natural causes 3) intermediate death with terminal phase of 1-24 hours 4) slow death after long illness.*
