# Supplementary material for: Diabetes risk loci-associated pathways are shared across metabolic tissues
Source: BMC Genomics. 2022 May 14;23:368. doi: 10.1186/s12864-022-08587-5 (PMC9107144; doi:10.1186/s12864-022-08587-5)
Supplement: Supplementary file 4 — Additional file 4: Table S3. Protein, metabolite and expression QTLs associated with rs1260326. [file 12864_2022_8587_MOESM4_ESM.docx]

**Table S3 Protein, metabolite and expression QTLs associated with rs1260326**

| **QTL** | **Protein** | **EA** | **OA** | **Beta** | **SE** | **P value** | **Cis/Trans** | **H4 PP** | **Tissue** |
| --- | --- | --- | --- | --- | --- | --- | --- | --- | --- |
| pQTL | IGFBP1 | T | C | 0.14 | 0.02 | 2.42·10^-13^ | Trans | 0.873 | Plasma |
|  | KLKB1 | T | C | 0.12 | 0.02 | 2.13·10^-10^ | Trans | 4.23·10^-46^ | Plasma |
| mQTL | Ala | T | C | 0.11 |  | 7.31·10^-19^ |  |  | Plasma |
|  | VLDL_TG | T | C | 0.09 |  | 1.97·10^-13^ |  |  | Plasma |
|  | S_VLDL_TG | T | C | 0.09 |  | 4.81·10^-13^ |  |  | Plasma |
|  | Serum_TG | T | C | 0.09 |  | 6.02·10^-13^ |  |  | Plasma |
|  | M_VLDL_TG | T | C | 0.09 |  | 9.45·10^-13^ |  |  | Plasma |
|  | XL_VLDL_TG | T | C | 0.09 |  | 3.97·10^-12^ |  |  | Plasma |
|  | L_VLDL_TG | T | C | 0.09 |  | 4.63·10^-12^ |  |  | Plasma |
|  | M_VLDL_P | T | C | 0.09 |  | 5.28·10^-12^ |  |  | Plasma |
|  | L_VLDL_P | T | C | 0.09 |  | 7.24·10^-12^ |  |  | Plasma |
|  | L_VLDL_PL | T | C | 0.09 |  | 8.87·10^-12^ |  |  | Plasma |
|  | TG_PG | T | C | 0.08 |  | 9.52·10^-12^ |  |  | Plasma |
|  | M_VLDL_PL | T | C | 0.08 |  | 9.78·10^-12^ |  |  | Plasma |
|  | XL_VLDL_P | T | C | 0.08 |  | 1.16·10^-11^ |  |  | Plasma |
|  | VLDL_D | T | C | 0.08 |  | 2.24·10^-11^ |  |  | Plasma |
|  | S_VLDL_P | T | C | 0.08 |  | 5.07·10^-11^ |  |  | Plasma |
|  | XL_VLDL_PL | T | C | 0.08 |  | 9.37·10^-11^ |  |  | Plasma |
|  | MUFA | T | C | 0.08 |  | 3.37·10^-10^ |  |  | Plasma |
|  | XS_VLDL_TG | T | C | 0.08 |  | 1.56·10^-9^ |  |  | Plasma |
|  | S_VLDL_PL | T | C | 0.08 |  | 1.82·10^-9^ |  |  | Plasma |
|  | M_VLDL_L | T | C | 0.09 |  | 2.94·10^-9^ |  |  | Plasma |
|  | L_VLDL_FC | T | C | 0.09 |  | 4.03·10^-9^ |  |  | Plasma |
|  | XL_VLDL_L | T | C | 0.08 |  | 1.05·10^-8^ |  |  | Plasma |
|  | Gp | T | C | 0.07 |  | 1.06·10^-8^ |  |  | Plasma |
|  | XXL_VLDL_TG | T | C | 0.07 |  | 1.09·10^-8^ |  |  | Plasma |
| eQTL | *SNX17* | T | C | -0.32 | 0.03 | 1.29·10^-32^ |  | 0.483 | Muscle_Skeletal |
|  | *NRBP1* | T | C | -0.19 | 0.03 | 7.50·10^-12^ |  | 0.985 | Adipose_Subcutaneous |
|  | *FNDC4* | T | C | 0.21 | 0.03 | 4.15·10^-11^ |  | 0.041 | Thyroid |
|  | *NRBP1* | T | C | -0.11 | 0.02 | 6.44·10^-10^ |  | 0.995 | Whole_Blood |
|  | *NRBP1* | T | C | -0.15 | 0.03 | 9.26·10^-9^ |  | 0.988 | Colon_Transverse |
|  | *GCKR* | T | C | 0.22 | 0.04 | 1.84·10^-8^ |  | 0.058 | Thyroid |
|  | *ATRAID* | T | C | 0.19 | 0.03 | 5.56·10^-8^ |  | 0.975 | Colon_Transverse |
|  | *NRBP1* | T | C | -0.15 | 0.03 | 9.69·10^-7^ |  | 0.990 | Adipose_Visceral_Omentum |
|  | *PPM1G* | T | C | -0.15 | 0.03 | 1.14·10^-6^ |  | 0.973 | Pancreas |
|  | *NRBP1* | T | C | -0.21 | 0.04 | 2.18·10^-6^ |  | 0.980 | Small_Intestine_Terminal_Ileum |
|  | *GPN1* | T | C | -0.12 | 0.03 | 2.53·10^-6^ |  | 0.106 | Muscle_Skeletal |
|  | *KRTCAP3* | T | C | 0.19 | 0.04 | 2.91·10^-6^ |  | 0.797 | Muscle_Skeletal |
|  | *C2orf16* | T | C | 0.13 | 0.03 | 6.17·10^-6^ |  | 0.836 | Thyroid |
|  | *KRTCAP3* | T | C | 0.16 | 0.04 | 1.32·10^-5^ |  | 0.394 | Whole_Blood |
|  | *ATRAID* | T | C | 0.09 | 0.02 | 1.61·10^-5^ |  | 0.975 | Thyroid |
|  | *AC074117.10* | T | C | -0.17 | 0.04 | 2.87·10^-5^ |  | 0.960 | Pancreas |
|  | *PPM1G* | T | C | -0.07 | 0.02 | 3.45·10^-5^ |  | 0.948 | Adipose_Subcutaneous |
|  | *PPM1G* | T | C | -0.06 | 0.01 | 5.93·10^-5^ |  | 0.955 | Muscle_Skeletal |
|  | *AC074117.10* | T | C | -0.11 | 0.03 | 7.91·10^-5^ |  | 0.808 | Adipose_Visceral_Omentum |
|  | *AC074117.10* | T | C | -0.09 | 0.02 | 2.13·10^-4^ |  | 0.926 | Thyroid |
|  | *ATRAID* | T | C | 0.08 | 0.02 | 2.15·10^-4^ |  | 0.952 | Adipose_Subcutaneous |

*EA, effect allele; OA, other allele.*
