# Supplementary material for: Diabetes risk loci-associated pathways are shared across metabolic tissues
Source: BMC Genomics. 2022 May 14;23:368. doi: 10.1186/s12864-022-08587-5 (PMC9107144; doi:10.1186/s12864-022-08587-5)
Supplement: Supplementary file 5 — Additional file 5: Table S4. Protein and expression QTLs associated with rs474513. [file 12864_2022_8587_MOESM5_ESM.docx]

**Table S4 Protein and expression QTLs associated with rs474513**

| QTL | Protein | EA | OA | Beta | SE | P value | H4 PP | Cis/Trans | Tissue |
| --- | --- | --- | --- | --- | --- | --- | --- | --- | --- |
| pQTL | LPA | A | G | 0.228 | 0.018 | 8.27E-37 | 1.41·10^-107^ | Cis | Plasma |
| eQTL | SLC22A3 | A | G | -0.53 | 0.07 | 2.23E-12 | 0.096 | Cis | Liver |
|  | SLC22A3 | A | G | 0.18 | 0.03 | 4.67E-08 | 0.027 | Cis | Adipose_Subcutaneous |
|  | SLC22A3 | A | G | 0.18 | 0.04 | 8.66E-07 | 0.072 | Cis | Thyroid |
|  | SLC22A3 | A | G | 0.15 | 0.03 | 1.09E-06 | 0.007 | Cis | Adipose_Visceral_Omentum |
|  | LPA | A | G | 0.29 | 0.07 | 1.29E-05 | 0.067 | Cis | Liver |

*PP, posterior probability; EA, effect allele; OA, other allele; SE, standard error*
